# Processes and Recommendations for Creating mHealth Apps for Low-Income Populations

**DOI:** 10.2196/mhealth.6510

**Published:** 2017-04-03

**Authors:** Laura Siga Stephan, Eduardo Dytz Almeida, Raphael Boesche Guimaraes, Antonio Gaudie Ley, Rodrigo Gonçalves Mathias, Maria Valéria Assis, Tiago Luiz Luz Leiria

**Affiliations:** ^1^ Instituto de Cardiologia Fundação Universitária de Cardiologia Porto Alegre, RS Brazil; ^2^ MR Apps Porto Alegre, RS Brazil; ^3^ Aalto University Helsinki Finland

**Keywords:** mHealth, atrial fibrillation, low-income population

## Abstract

**Background:**

Mobile health (mHealth) apps have shown to improve health indicators, but concerns remain about the inclusion of populations from low- and medium-income countries (LMIC) in these new technologies. Atrial fibrillation (AF) is a chronic condition with a challenging management. Previous studies have shown socioeconomic differences in the prescription of anticoagulant treatment and shared decision strategies are encouraged to achieve better outcomes. mHealth can aid both doctors and patients in this matter.

**Objective:**

We describe the development of an mHealth app (*aFib*) idealized to aid shared decision between doctor and patient about anticoagulation prophylaxis in AF in a low-income and low-literacy population in Brazil. On the basis of our research, we suggest the processes to be followed when developing mHealth apps in this context.

**Methods:**

A multidisciplinary team collected information about the target population and its needs and detected the best opportunity to insert the app in their current health care. Literature about the subject was reviewed and important data were selected to be delivered through good navigability, easy terminology, and friendly design. The app was evaluated in a multimethod setting.

**Results:**

The steps suggested to develop an mHealth app target to LMIC are: (1) characterize the problem and the target user, (2) review the literature, (3) translate information to knowledge, (4) protect information, and (5) evaluate usability and efficacy.

**Conclusions:**

We expect that these recommendations can guide the development of new mHealth apps in LMIC, on a scientific basis.

## Introduction

Mobile device usage has increased dramatically over the years and recent data has shown that 64% of adults in the United States own a mobile phone. About 62% of mobile phone owners have used their phones in the past year to look up information about a health condition [1], often using software programs, or “apps” of mobile health (mHealth) [2]. In 2016, there were around 3.2 billion of downloads of mHealth apps around the globe [3]. Apps have shown to improve treatment adherence [4] and risk factors control [2,5] for chronic diseases. However, there are concerns about the proper inclusion of populations from low- and medium-income countries (LMIC) in this new health care resource [2,6]. In Brazil, for instance, the current number of mobile devices in use is around 168 million [7], but most of these are basic phones with poor or no Internet access, which let their users relegated to the sidelines of this technology.

One of the diseases that can have its management improved with the help of mHealth is atrial fibrillation (AF), a common arrhythmia largely associated with stroke risk [8]. Anticoagulation therapy reduces this risk [9], but it can be challenging due to associated comorbidities and bleeding risk [10]. Hence, it is paramount that decisions about AF treatment be shared between health care providers and patients [11]. It is recognized that there are socioeconomic differences in AF treatment. Among different socioeconomic strata, those with lower income were less frequently prescribed anticoagulants [12].

mHealth apps targeted to low-resource settings must be evidence-based, efficient, safe, and tailored to the users and their needs. To achieve these goals and maintain usability, special focus should be given to four specific themes: interface design, feedback, navigation, and terminology [13]. However, most of these recommendations are not followed and systematically evaluated in a real-world setting before the go-live phase [2,13].

In this paper, we describe the development of an mHealth app idealized to aid shared decision about anticoagulation in AF in a low-income and low-educational level population in Brazil. On the basis of our research and results, we suggest processes and recommendations to be followed when developing mHealth apps adapted to use in LMIC.

## Methods

### aFib Study Overview

We developed an mHealth prototype, named *aFib*, targeted for patients with AF and their doctors. The main purpose was to facilitate shared decision on anticoagulation therapy during clinical appointment.

Our study population comprised patients with AF followed at the outpatient anticoagulation clinic of our institution. *Instituto de Cardiologia do Rio Grande do Sul* is a hospital specialized in treating heart diseases, supported by the Brazilian public health system. Every month, around 700 patients with AF are followed by the anticoagulation clinic and the majority of them have low-income and low-educational level. Previous studies showed that only half of these patients are adherent to therapy or within the therapeutic range of international normalized ratio (INR) [14,15], showing that existing strategies are not effective.

### App Development Process

The *aFib* team comprised a cardiac electrophysiologist with expertise in AF, a clinical cardiologist, a software developer, and a designer. After a comprehensive literature review to support all information provided by the app, we used the behavioral intervention technology (BITs) model to ensure that the development process was systematic and replicable [16]. Our process comprised the following steps:

- Establishing the clinical aims (WHY): (a) increase knowledge about AF, stroke risk, bleeding risk, and anticoagulants [17,18] and (b) promote a shared decision about the therapy [19].
- Selecting behavioral strategies to increase knowledge in low-income populations and aid shared decision (HOW): (a) education, (b) dialog with their doctors, and (c) motivation enhancement.
- Designing app elements to deliver selected behavioral strategies (WHAT): (a) a 1.5-min long educational video about how AF can cause stroke; (b) a one-screen-only calculator for the two most recommended risk scores for stroke and bleeding [11]; (c) a screen with pictograms for a better understanding of the scores by poor literacy patients; and (d) an short message service (SMS) system to continue delivering information to users about their disease and treatment.
- Defining when and under what conditions the app will be used (WHEN): as many of our patients have basic handsets that can accommodate only voice and SMS text messaging, our option was to provide this first mHealth interaction during clinical consultation, using the physician’s mobile device.

All information collected could be saved and reviewed later. This was motivated by 3 reasons: (1) risk factors often change over time, which means that risk stratification must be updated and recalculated; (2) to allow a staff nurse to collect primary data that could be expanded and modified during the physician’s appointment, providing greater ease of adoption of the app in the primary care setting; and (3) to enable data transfer to other devices, since we expect that our target population will likely migrate to mobile phones in the following years.

*aFib* app prototype was delivered in Portuguese. Terminology was adapted to both patients and health professionals according to whom the information was directed to. Language in the educational video, in the pictograms, and in the summarized information about medications was directed to low-literacy patients, whereas language of the risk scores and leaflets was directed to their caregivers. Graphic design was developed to be clear and intuitive, with few but meaningful graphics and a color code to highlight risks and benefits of the treatment. The first version was designed to Android tablets with 10.1-inch screen to improve reading since the majority of patients with AF are elderly and may have vision impairment. Security and privacy were assured through unique ID authentication, and data transfer to central database was done using a Transport Layer Security (TLS) with 128-bit encryption method. A privacy policy was presented at the beginning of data collection with appropriate information, purpose, and user rights. See Multimedia Appendix 1 for main screens of *aFib*.

Three clinical cardiologists, 2 cardiac electrophysiologists, 1 medicine student, and 20 patients evaluated *aFib* in the pilot phase and gave feedback about perceived ease of use, perceived usefulness (two statements with a 5-point Likert scale), relevancy of content, navigation, terminology, interactivity, attractiveness, learnability (through a pre- and posttest questionnaire about the disease and treatment), and conflict about decision process (measured by a decisional conflict scale) [20]. Written informed consent was obtained from all participants, and the Institutional Ethics Committee approved the study.

After adjustments that were made based on these feedbacks, *aFib* is being currently evaluated in a randomized clinical trial with the target population described, with short- and long-term outcomes previously established (improve knowledge, treatment adherence, and maintenance of adequate levels of anticoagulation and cost-effectiveness of this strategy).

The main challenges in the development process were (1) in the app contents, to summarize information in a way that would maximize knowledge acquisition and maintain patient’s attention; (2) in the design, to minimize screens and to translate the percentages of risk in graphic information understandable by low-literacy users; (3) in the technical area, to level the knowledge of the development team in order to permit brainstorming, that is, to provide the developer and the designer with the medical information needed to understand the app context and, at the same time, to provide physicians with the technical information essential for structuring the app.

## Results

### Key Recommendations

**Figure 1:**
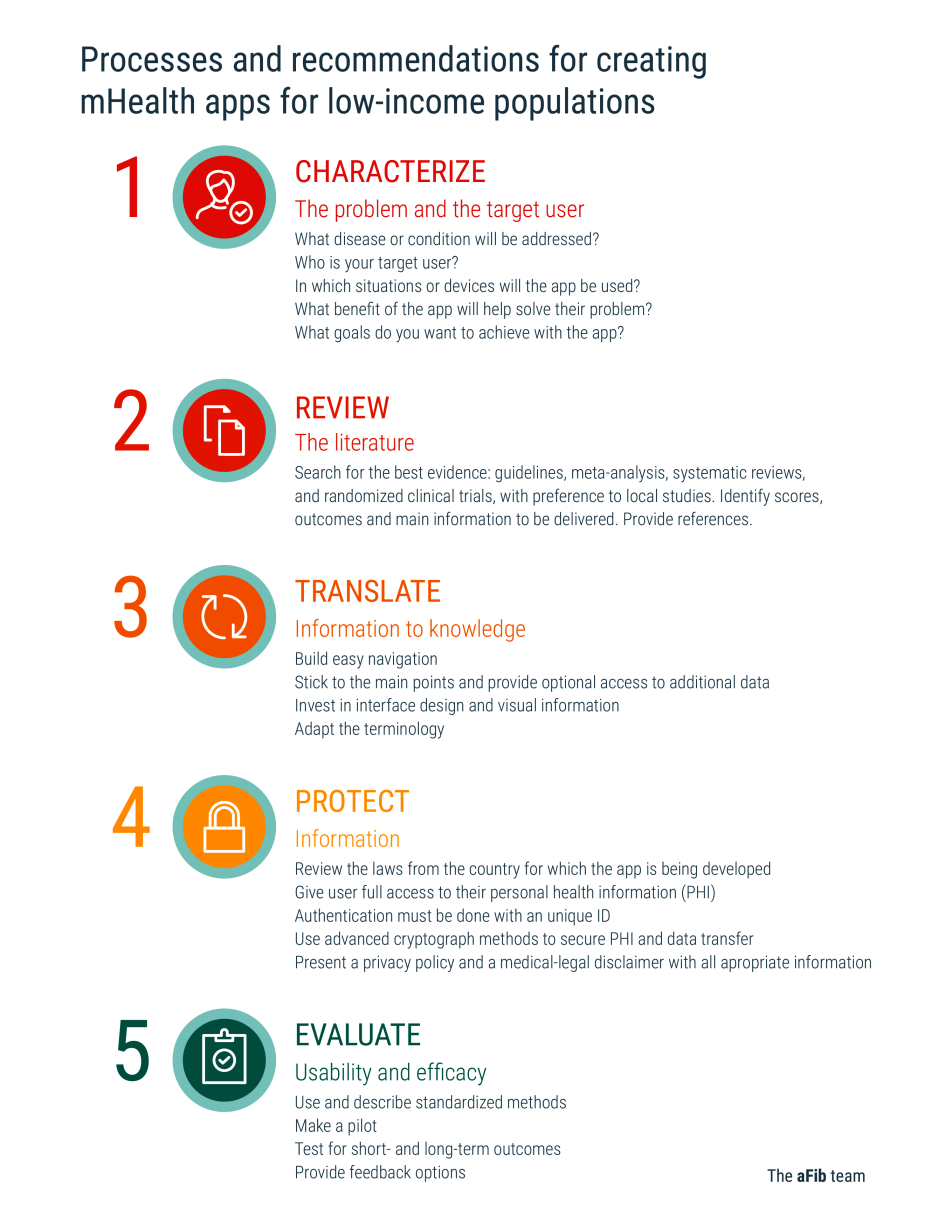
Processes and recommendations for creating mHealth apps for low-income populations, based on the aFib experience.

On the basis of this experience, we created a short guide to the development of mHealth apps (Figure 1), suitable for use in other oncoming mHealth apps for use in LMIC.

### Characterize the Problem and the Target User

A solid project starts with understanding who the users are and what problem do the app want to solve. If the subject in question is too broad, try to approach a key problematic aspect, like knowledge gaps, difficult choices, or complex treatments.

An mHealth app may be directed to patients or their relatives, health care professionals, or even to healthy people looking for disease prevention. It is important to understand not only the socioeconomic background and educational level of the target population, but also their desires and doubts. What is their most pressing issue? Where do they get their information? What benefit of the app will help solve their problem? All these questions must be answered in order to develop a user-centered mHealth app. It is important to think also about the Internet connectivity and multimedia capability of the device in which the app will be accessed. In the United States, low-income groups are now using mobile phones as their primary method for Internet access [21]. In other countries, it might be desirable that the content can be downloaded to be accessed offline. Usability has to be planned foreseeing the worst scenario.

### Review the Literature

Citizens from LMIC can have inconsistent access to their health systems, and an mHealth app can be a great opportunity to reach these populations. Thus, it should provide the best available evidence about the problem: guidelines, meta-analysis, systematic reviews, and randomized clinical trials will be the most helpful, with preference to local guidelines. Identify risk scores, outcomes, and the core information to be delivered. The references should be provided in order to aid users to make their own judgment on its reliability.

Ascertain the prevalence, incidence, and other important measures of impact, effect, and association of the problem in the target population. This would help to justify the app contents and/or interventions and establish outcomes.

If the subject had been addressed before by other apps, then identify which knowledge gaps are still blank and how to fill local population needs.

### Translate Information to Knowledge

Once the problem has been defined globally and the content is evidence-based, the main challenge is to transform a huge amount of data in best quality and well-presented information, tailored to the user. Low literacy can be a barrier to the adoption of usual solutions. The 4 topics that should be kept in mind to better achieve the goal of transmitting knowledge are discussed below.

#### Build Easy Navigation

This refers to the way a user navigates throughout the app to complete tasks. If it is expected that the app will be used in a context of frequent interruptions, multitasking, and increased cognitive load, bad navigation may result in errors of attention and attribution [22]. Icons, tab views, and buttons should be easily recognizable [13]. It is possible to reduce cognitive load by creating steps, unifying scores, and gathering information to optimize time and minimize the number of screen switching. Screen size must also be taken into account since the smaller screen of a mobile phone will need more screen switches compared with the larger screen of a tablet.

#### Stick to the Main Points

There are several ways to reduce the amount of information, but any such reduction is also limited by the potential loss of critical information [22]. Main information must be highlighted, but the app should provide ways to access more complete references (eg, through hyperlinks or information buttons). Although an app is a pull technology, additional reinforcement can be achieved by employing push technology like SMS. Push and pull technology is widely used in LMIC; some examples are the improvement of early infant diagnosis of human immunodeficiency virus (HIV) in Zambia [23] and health education through messages in Benin, Malawi, and Uganda [24]. Personalized messages are seen as the most easily implemented and most effective strategy in changing patient behavior [3].

#### Invest in Interface Design

This theme refers to the design and layout, including consistency, location of icons, functions on each screen, font, color, density, placement, and images [13]. People learn better from visual information [25]. In a low-literacy scenario, cartoons can be used to simplify the message. The right images can improve comprehension, trigger emotions, and stick in long-term memory, but the incorrect use can deter instructiveness. mHealth users’ main complaints about app design are related to visibility (as well as small screen space), hard to read fonts, lack of color coding, and poor graphic displays [26]. An effective visual communication include (1) appropriate and legible typography, (2) use of no more than five colors in the layout, (3) simple, easy to understand, and universal iconography, (4) spare use of callouts to highlight only key information, (5) significant negative space between messages, (6) use of illustration only if it enhances the content, and (7) maintain a logical hierarchy of the contents [27].

#### Adapt the Terminology

Terminology reflects the user’s ability to identify with and understand the language used within the app [13]. Linguistic and cultural customization of health-related contents improve the involvement of low-income populations and the first step of this process is related to the first recommendation of this tutorial, that is, to characterize the target user [28]. Apps aimed at poor literacy populations must adopt health vocabulary that they use routinely. It can also be useful if the correct terminology is mentioned, between parentheses, for example, for learning purposes. Consultants that represent the target user’s population should revise all contents in the pilot evaluation. Important questions that can be asked are (1) What was the main idea? (2) Was it easy to read or understand? (3) Would you change any term to improve understanding?

### Protect Information

Security and privacy in mHealth apps is a vast subject, and laws regulating these aspects vary widely between countries. A recent review addressed this issue and suggested minimum requirements for developers [29], summarized here:

- The user should be able to control access to their personal health information (PHI) at any moment.
- Authentication must be done with a unique ID.
- Use advanced encryption standard (AES) to encrypt PHI, with a cryptographic key of at least 128 bits.
- Before PHI is collected, present a privacy policy with all appropriate information, including data retention, purpose, and user rights.
- Data transfer should be done with TLS with 128-bit encryption methods or virtual private networks (VPNs).
- Cryptographic methods must be used in securing the body sensor networks for authentication and key distribution.
- In case of a PHI breach, the competent authority and the affected user must be notified as soon as possible (1-3 days) and possible consequences must be relieved.

We strongly recommend a comprehensive review of the laws from the country for which the app is being developed since these minimum requirements can be insufficient in some cases.

A built-in medical-legal disclaimer with the terms of use agreement is of special interest if the app is widely available for download by doctors and patients, to help clarify the publisher’s responsibilities and thereby reduce the legal risks associated with the use.

### Evaluate Usability and Efficacy

Recent studies about mHealth usability suggest the use of a multimethod approach and standardized methods and tools in mHealth evaluations, which can result in a more comprehensive identification of usability issues, more specific redesign recommendations, and better reproducibility of results across studies. For users, improved mHealth design and usability could result in improved interactions, greater use of mHealth apps, and perhaps even increased adherence to suggested interventions and therapeutic [30].

To date, there is a paucity of published studies on the efficacy of mobile phone apps for health promotion in low-income populations. More research and evaluation is necessary about both internal and external validity and sustained health outcomes [31].

Internal validity of mHealth apps for low-income populations can be threatened by selection bias (eg, participants with sociocultural characteristics that do not represent the target population), instrument changes (eg, version updates of the app), regression toward the mean (eg, in knowledge evaluation of low-literacy participants), between other factors. To improve and standardize evaluation reports of mHealth interventions, a validated framework like CONSORT-EHEALTH [32] or RE-AIM [33] is recommended. The following are the steps that can be followed to validate an app.

#### Make a Pilot

For formative usability tests, 5-8 users are able to detect 80-85% of usability problems [34]. A pilot study of the app is imperative to adjust design and navigability based on user-centered data.

#### Test for Short-Term Outcomes

First real-world trial of the app should analyze short-term outcomes, such as knowledge increase, user satisfaction, behavioral changes, and improvements in validated tests, scores, or modifiable risk factors.

#### Test for Long-Term Outcomes

If the proposed new health care tool can act positively on factors mentioned above, it could possibly improve long-term outcomes as well. The ideal scenario is to test the mHealth app in a randomized trial with preestablished clinical outcomes, and to evaluate cost-effectiveness when compared with existing strategies. Burke et al [2], on its recent review about current science on mHealth, have proposed some interesting questions that future mHealth apps will have to answer: Does the product work best when used in certain settings or among specific patient groups? Does the app potentiate impact when it is combined with other traditional interventions? In what cases can the findings be generalized among similar technologies in the class? Are the effects seen durable? Are there any unintended consequences associated with the device and program in which it is used? [2] We need to embrace the challenge of producing this needed evidence.

#### Provide Feedback Options

After go-live phase, it is important that users can continue evaluating all of the aforementioned aspects. All app stores have this option, but users can be stimulated to give their opinions and a customer service should be organized to answer main doubts.

## Discussion

### Principal Findings

Achieving success in health prevention and treatment in LMIC is challenging and require approaches and tools that (1) have proven clinical benefit, (2) can be scaled to reach a global population and (3) are affordable. Mobile technologies provide a potential platform to facilitate these needs [35].

mHealth is empowering individuals to assume a more active role in monitoring and managing their chronic conditions and therapeutic regimens, as well as their health and wellness [2]. But, this new health tool needs to be adapted to local language and reality [6].

This tutorial was based on the experience acquired while developing the app “aFib,” and describes processes and recommendations to aid developers who are interested in creating or adapting mHealth apps to low-income populations. A problem-targeted and user-centered strategy appears to be a logical trend to the development of these apps. A comprehensive review of literature must be the base of the project. The best efforts should be made to translate information to knowledge and it should be kept in mind that good navigability, terminology, and interface design can help on this task. Protection of user information is essential, and local laws on the matter should be studied beforehand. Usability and efficacy must always be evaluated in a variety of scenarios.

This tutorial has several limitations. First, the literature about mHealth is increasing quickly and new models are being tested every day to improve the development process, which can soon overwhelm the models we used here. Second, it is likely that a single experience cannot provide insight about every step of this process. Third, other diseases or conditions can present with different challenges related to low-income populations. Finally, the characteristics of low-income populations vary widely between countries, with cultural and social differences that may need distinct adaptations.

Despite these limitations, we believe that it can encourage the development and evaluation of mHealth apps in LMIC. mHealth is a low-cost strategy and appear to be an appreciated, easy-to-use, and promising aid to improve knowledge and engage individuals from LMIC in the management of their illness, supporting healthy behavior change and potentially improving population health.

### Conclusions

As mobile technologies become increasingly ubiquitous, apps adapted to local use in low-income populations are needed. This tutorial expects to stimulate the development of mHealth apps for low-income populations, on a scientific base. Future work should address other possible ways to reach this special group and the extent to which this new health resource will affect their health care.

## Appendix

Multimedia Appendix 1 Main screens of aFib app.

## Abbreviations

AES: advanced encryption standard
AF: atrial fibrillation
apps: applications
BITs: behavioral intervention technology
HIV: human immunodeficiency virus
ID: identification
LMIC: low- and medium-income countries
mHealth: mobile health
PHI: personal health information
INR: international normalized ratio
SMS: short message service
TLS: Transport Layer Security
VPN: virtual private network
