# Supplementary figures and images for: Processes and Recommendations for Creating mHealth Apps for Low-Income Populations
Source: JMIR Mhealth Uhealth. 2017 Apr 3;5(4):e41. doi: 10.2196/mhealth.6510 (PMC5394264; doi:10.2196/mhealth.6510)

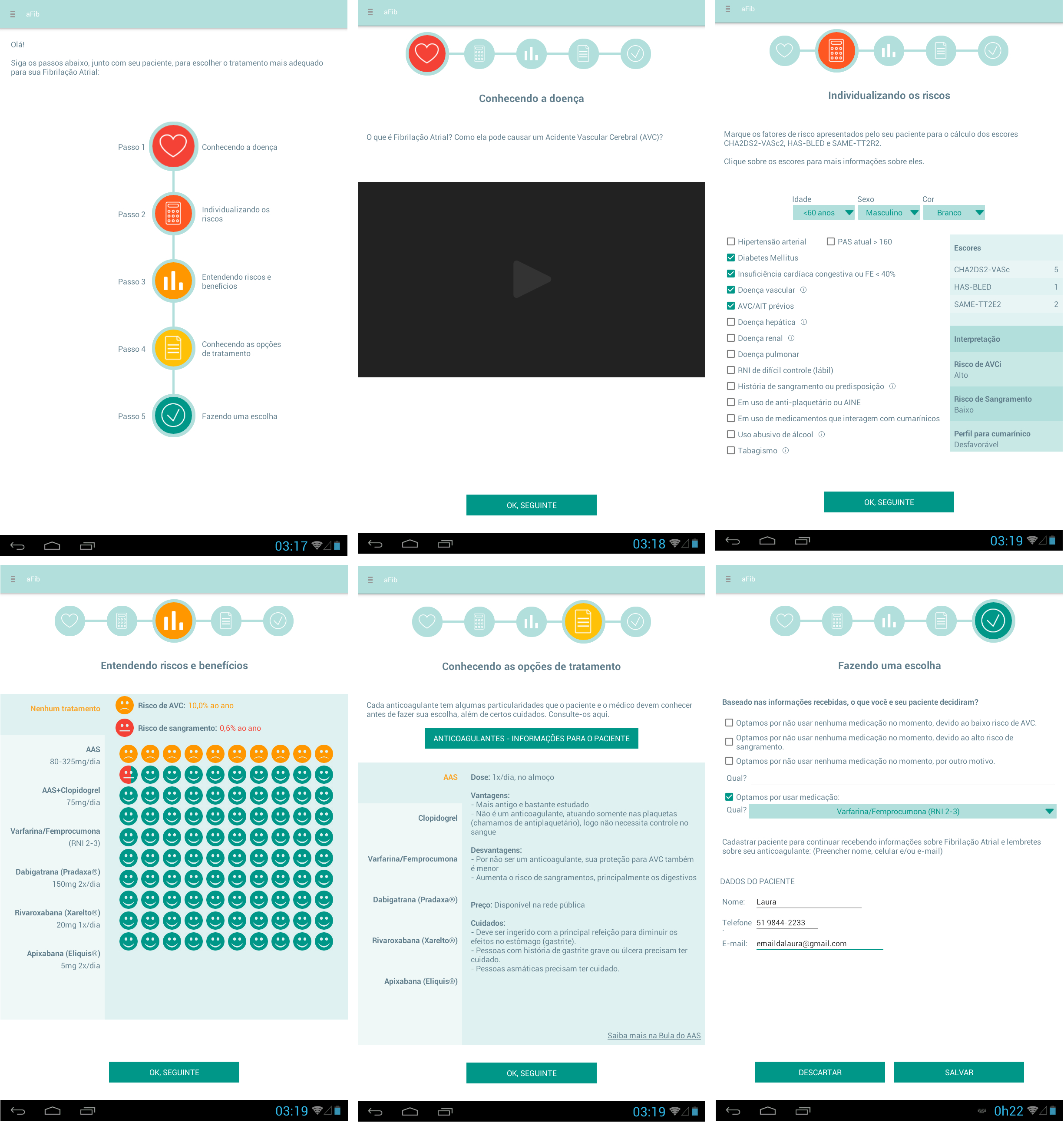

Supplement: Multimedia Appendix 1 [file mhealth_v5i4e41_app1.jpg]
